# Combined Treatment of Cinobufotalin and Gefitinib Exhibits Potent Efficacy against Lung Cancer

**DOI:** 10.1155/2021/6612365

**Published:** 2021-03-20

**Authors:** Youxi Han, Ronghui Ma, Guolei Cao, Hao Liu, Lili He, Le Tang, Hong Li, Qin Luo

**Affiliations:** ^1^Department of Breast Radiotherapy, The Affiliated Tumor Hospital of Xinjiang Medical University, Urumqi 830000, China; ^2^Department of Respiratory Neurology, The Affiliated Tumor Hospital of Xinjiang Medical University, Urumqi 830000, China; ^3^Department of Infection, The Affiliated Tumor Hospital of Xinjiang Medical University, Urumqi 830000, China

## Abstract

This study aimed to evaluate the efficacy of cinobufotalin combined with gefitinib in the treatment of lung cancer. A549 cells were treated with gefitinib, cinobufotalin, or cinobufotalin plus gefitinib. MTT assay, annexin-V/PI staining and flow cytometry, TUNEL staining, DCFH-DA staining, Western blot, and real-time RT-PCR were performed to investigate the synergistic inhibitory effect of cinobufotalin combined with gefitinib on the growth of A549 cells. Results showed that cinobufotalin synergized with gefitinib displayed inhibited cell viability and enhanced apoptosis in the combination group. Cinobufotalin combined with gefitinib induced a significant enhancement in reactive oxygen species (ROS) production accompanied by cell cycle arrest in the S phase arrest, characterized by upregulation of p21 and downregulation of cyclin A, cyclin E, and CDK2. Besides, cinobufotalin plus gefitinib downregulated the levels of HGF and c-Met. In summary, cinobufotalin combined with gefitinib impedes viability and facilitates apoptosis of A549 cells, indicating that the combined therapy might be a new promising treatment for lung cancer patients who are resistant to gefitinib.

## 1. Introduction

Lung cancer is one of the most typical cancers worldwide, with more than 2 million new cases and over 1.7 million deaths each year [1], of which nonsmall cell lung cancer (NSCLC) accounts for 85% of all lung cancers [2]. EGFR mutations are known to be carcinogenic driver mutations that occur in 10–44% of lung adenocarcinomas. In recent decades, apart from traditional treatments such as surgery, chemotherapy, and radiotherapy, epidermal growth factor receptor- (EGFR-) tyrosine kinase inhibitors (TKIs) have been widely used in the treatment of NSCLC patients [3]. In addition to enhancing progression-free survival of patients, EGFR-TKIs are less toxic than chemotherapy [4]. Previous studies have reported that gefitinib, the first-generation of EGFR-TKIs, is effective against tumors with EGFR activation mutations including EGFR L858R and EGFR del-19 [5, 6]. Nevertheless, even if NSCLC patients harboring gene mutations initially respond to gefitinib, drug resistance inevitably develops [7].

Toschi et al. believed that NSCLC resistance to first-generation EGFR-TKIs was related to aberrant cell-mesenchymal epidermal transformation factor (c-Met) activity [8]. c-Met has been proven to be the only high proreceptor that binds to the hepatocyte growth factor (HGF). Abnormal c-Met activity in NSCLC can be elevated by MET gene mutation and amplification or upregulated HGF expression. After the binding of HGF to c-Met, autophosphorylation of c-Met activates a variety of intracellular signaling pathways, promoting tumor progression, invasion, and metastasis [9]. In lung cancer with aberrant c-Met activity leading to gefitinib resistance, sensitivity to gefitinib may be restored through suppression of c-Met signaling [7].

Currently, traditional Chinese medicine has attracted much attention because of its potent application in anticancer treatment. Cinobufotalin (huachansu) is extracted from the skin secretions of *Bufo gargarizans* , with benefits of detoxification, promoting blood circulation and removing blood stasis [10]. Several studies have shown that cinobufotalin can regulate immune function and promote apoptosis of tumor cells [11]. Cinobufotalin combined with chemotherapeutic agents has shown potent anticancer effects in a variety of cancers, such as liver cancer, pancreatic cancer, lung cancer, and hepatocellular carcinoma [12]. However, it has not been determined whether cinobufotalin in combination with gefitinib can be used for the treatment of lung cancer.

Herein, this study evaluated the efficacy of cinobufotalin combined with gefitinib on the growth of human lung adenocarcinoma A549 cells, providing a new therapeutic therapy for the treatment of NSCLC.

## 2. Materials and Methods

### 2.1. Cell Culture and Drug Treatment

Human nonsmall cell lung adenocarcinoma A549 cell strain and normal human lung BEAS-2B cell strain were provided by the Third Affiliated Hospital of Xinjiang Medical University Cancer Institute. Cinobufotalin and gefitinib were purchased from Solarbio and Sinopharm, respectively. A549 and BEAS-2B cells were cultured in RPMI1640 medium containing 10% fetal bovine serum (Invitrogen), 100 U/mL penicillin, and 100 U/mL streptomycin at 37°C in a humidified atmosphere of 5% CO_2_ (pH 7.2–7.4). The cells at the logarithmic growth stage were divided into the control group (DMSO), gefitinib group (1, 5, 10, 20, and 40 *μ*mol/L), cinobufotalin group (0.005, 0.01, 0.05, 0.1, and 0.5 mg/mL), and cinobufotalin plus gefitinib group.

### 2.2. MTT Assay

The cell density of both A549 and BEAS-2B was adjusted to 5 × 10^4^/mL and inserted into a 96-well plate, followed by the addition of cell suspension 100 *μ*L. At 24, 48, and 72 h, MTT solution 10 *μ*L (Wuhan Biofavor Biotechnology Service Co., Ltd.) was added for further culture for 4 h, and the medium was sucked out. The formazan crystals were dissolved in DMSO 150 *μ*L. The blank pores were used as blank groups. The absorbance (OD) at 568 nm was measured by the microplate reader. The experiment was repeated three times.

### 2.3. Apoptosis and Cell Cycle Assays

A549 cells were inoculated in a 6-well plate at a density of 2 × 10^5^ cells per well, and cultured in an incubator of 5% CO_2_ at 37°C for 48 h. After incubation, cells were digested with 0.25% trypsin without EDTA and then rinsed with PBS twice and resuspended in the 500 *μ*L of binding buffer. Subsequently, cells were mixed with annexin-V-FITC 5 *μ*L and propidium iodide (PI) 5 *μ*L (Nanjing KeyGen Biotech Co., Ltd.) in the dark at room temperature for 5–15 min. Cell apoptosis rate and cell cycle were detected by flow cytometry (CytoFLEX, BECKMAN).

### 2.4. TUNEL Staining

TdT-mediated dUTP nick-end labeling (TUNEL) apoptosis detection kit was bought from Roche Applied Science Company. Cultured cells were immersed in 4% paraformaldehyde (pH7.4) solution at room temperature, followed by 0.1% PBS solution for 2 min. TUNEL reaction mixture 50 *μ*L was added in each group, and the slides were incubated at 37°C for 60 min in the dark. Notably, the control group was only added with luciferin-labeled dUTP solution 50 *μ*L. DAPI was added for incubation in dark for 5 min. Subsequently, the slides were sealed with an antifluorescent quenching agent, and the images were analyzed under a fluorescence microscope.

### 2.5. ROS Detection

A549 cells were incubated with 5 *μ*M of dichlorodihydrofluorescein diacetate (DCFH-DA) fluorescent probe for 20 min in the dark. DCFH-DA can be hydrolyzed by esterases to a nonfluorescent molecule 2,7-dichlorofluorescin and oxidized into fluorescent molecule 2,7-dichlorofluorescin in the presence of ROS. Then, the fluorescence emission was analyzed by a flow cytometer to detect the changes of ROS levels.

### 2.6. Western Blot

A549 cells were taken and washed with 3 mL of precooled PBS at 4°C. A 400 *μ*L of lysis buffer containing PMSF (100 mmol/L) was added for lysis, and the cells were centrifuged at 12000 r/min at 4°C for 5 min to extract the total protein. Protein concentration was determined by the bovine serum albumin protein quantification method, with a loading of 40 *μ*g total proteins per well. Subsequently, an equal amount of proteins for each group were separated by SDS-PAGE and transferred onto PVDF membranes. The membranes were then blocked in 5% skim milk in TBST and incubated overnight at 4°C with following primary antibodies: anti-Bax (rabbit polyclonal antibody, 00082363, Proteintech), anti-Bcl-2 (rabbit polyclonal antibody, 00083551, Proteintech), anti-cysteine-aspartic acid protease-3 (caspase-3, 15z0096, Affinity), anti-cyclin A (mouse polyclonal antibody, Proteintech), anti-cyclin E (rabbit polyclonal antibody, Proteintech), anti-P21 (rabbit polyclonal antibody, Proteintech), anti-cyclin-dependent kinase 2 (CDK2, rabbit polyclonal antibody, Proteintech), anti-MET (rabbit polyclonal antibody, Bioworld), anti-c-Met (rabbit polyclonal antibody, Proteintech), and anti-GAPDH (rabbit polyclonal antibody, AB-P-R001, Hangzhou Goodhere Co., Ltd.). The membranes were fully washed by TBST for 5-6 times and incubated with HRP-labeled sheep anti-rabbit secondary antibody (Wuhan Boster Biological Technology Co., Ltd.) at 37°C for 2 h. Finally, bands were detected via ECL luminescence kit. Bandscan was used to analyze the protein gray value and calculate the relative expression of the target protein.

### 2.7. Real-Time Fluorescence Quantitative PCR

Total RNA from cells was extracted using TRIzol reagent. A microspectrophotometer was used to measure the OD260 value, OD280 value, and OD260/OD280 ratio of RNA, as well as the purity and concentration of RNA. RNA quality was estimated based on the OD260/OD280 ratio (ratio range: 1.8–2.0). The concentration of the sample RNA was calculated according to the following formula: total RNA concentration (g/L) = OD260 × 40 × 10^−3^. The total RNA was reverse transcribed to cDNA, and the amplification and detection were performed by real-time quantitative PCR. The thermocycling conditions were set as follows: 50°C for 2 min and 95°C for 10 min, followed by 40 cycles at 95°C for 30 s and 60°C for 30 s. Final data were calculated with the 2−^△△^Ct method. The primer sequence is given in Table 1.

## 3. Statistical Analysis

SPSS 22.0 software was used for statistical analysis. Measurement data were expressed as mean ± standard deviation. The LSD *t*-test was used for comparison between every two groups, and analysis of variance (ANOVA) in repeated measurement was adopted for comparison among multiple groups. *P* < 0.05 was considered statistically significant.

## 4. Results

### 4.1. Combination of Cinobufotalin and Gefitinib Decreases the Viability of A549 Cells

**Figure 1:**
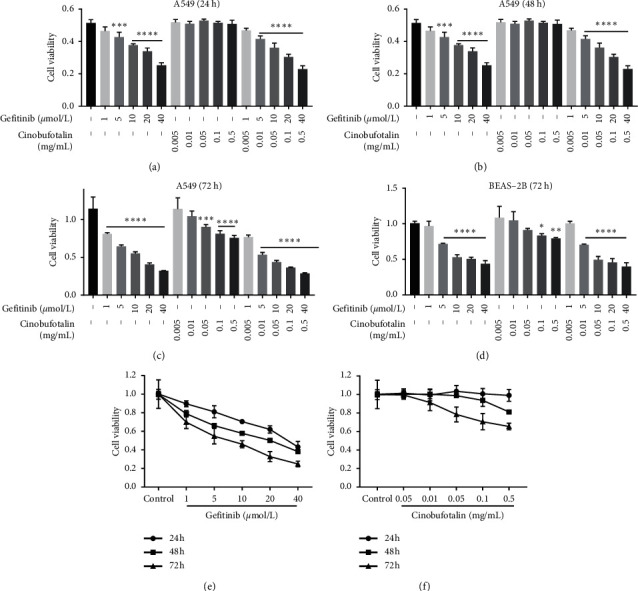
Cinobufotalin and gefitinib decreased A549 cell viability. MTT assay was performed to evaluate the inhibitory effect of gefitinib and cinobufotalin on A549 cells viability at (a) 24 h, (b) 48 h, and (c) 72 h either alone or in combination. (d) MTT assay was applied to assess the inhibitory function of gefitinib and cinobufotalin on BEAS-2B cell viability at 72 h either alone or in combination. The inhibitory effect of (e) gefitinib alone (1, 5, 10, 20, and 40 *μ*mol/L) and (f) cinobufotalin alone (0.005, 0.01, 0.05, 0.1, and 0.5 mg/mL) on A549 cell viability were also detected.  ^*∗*^*P* < 0.05,  ^*∗∗*^*P* < 0.01,  ^*∗∗∗*^*P* < 0.001, and  ^*∗∗∗∗*^*P* < 0.0001 vs. the control group (untreated cells). ^#^*P* < 0.05 vs. the gefitinib group at the corresponding drug concentration.

From the results of MTT assay, gefitinib alone (1, 5, 10, 20, and 40 *μ*mol/L) or combined with cinobufotalin significantly decreased the viability of A549 cells in a dose- and time-dependent manner (Figure 1). The half-maximal inhibitory concentration (IC50) of gefitinib was 24.53 *μ*mol/L at 24 h and 3.23 *μ*mol/L at 72 h in the combination group, which was lower than gefitinib alone (31.61 *μ*mol/L at 24 h and 6.61 *μ*mol/L at 72 h). However, the IC50 value of gefitinib alone in BEAS-2B cells was higher than that in A549 cells (IC50 = 17.12 *μ*M vs. IC50 = 6.61 *μ*M), indicating that the combined treatment of cinobufotalin and gefitinib was less toxic to normal cells (Figure 1(d)). Based on these results, gefitinib 40 *μ*mol/L and cinobufotalin 0.5 mg/mL were chosen for subsequent experiments.

### 4.2. Combination of Cinobufotalin and Gefitinib Induces A549 Cell Apoptosis

**Figure 2:**
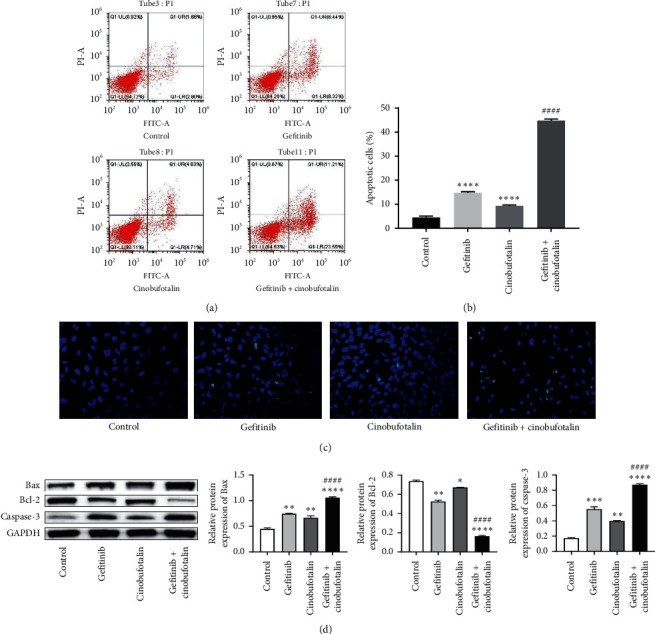
Combination of cinobufotalin and gefitinib induces A549 cells apoptosis. (a) A549 cells were treated with gefitinib 40 *μ*mol/L and cinobufotalin 0.5 mg/mL for 48 h either alone or in combination. Apoptotic rate of A549 cells was evaluated by annexin-V/PI staining and flow cytometry. (b) The percentage of apoptotic cells in the treatment groups was quantified. (c) TUNEL staining of A549 cells. A549 cells were treated with gefitinib 40 *μ*mol/L and cinobufotalin 0.5 mg/mL either alone or in combination and subsequently stained with TUNEL (magnification ×200). Green, apoptotic cells; blue, nucleus. (d) Western blot analysis of cell apoptosis-related proteins in A549 cells treated with gefitinib, cinobufotalin, or cinobufotalin + gefitinib.  ^*∗*^*P* < 0.05,  ^*∗∗*^*P* < 0.01,  ^*∗∗∗*^*P* < 0.001, and  ^*∗∗∗∗*^*P* < 0.0001 vs. the control group; ^####^*P* < 0.0001 vs. the gefitinib group.

We detected the A549 cell apoptosis under different treatments via flow cytometry and TUNEL assay. The apoptosis rate of A549 cells increased from 4.46% ± 0.65% in control to 14.76% ± 0.48%, 9.34% ± 0.37%, and 44.8% ± 0.62% with treatment of gefitinib 40 *μ*mol/L, cinobufotalin 0.5 mg/mL, and gefitinib plus cinobufotalin, respectively (Figures 2(a) and 2(b)). Subsequently, TUNEL assay was performed to further confirm the apoptotic effect of the combination therapy. Compared with the control group, cell apoptosis of each experimental group was remarkably elevated. In addition, the apoptosis rate of A549 cell in the combined treatment group was higher than that of the gefitinib group and cinobufotalin group (Figure 2(c)). In Western blot, the expression of Bax and caspase-3 in the A549 cells treated with gefitinib plus cinobufotalin was enhanced, while Bcl-2 expression was significantly downregulated (all *P* < 0.0001) (Figure 2(d)).

### 4.3. Combination of Cinobufotalin and Gefitinib Inhibits A549 Cell Cycle in the S Phase

**Figure 3:**
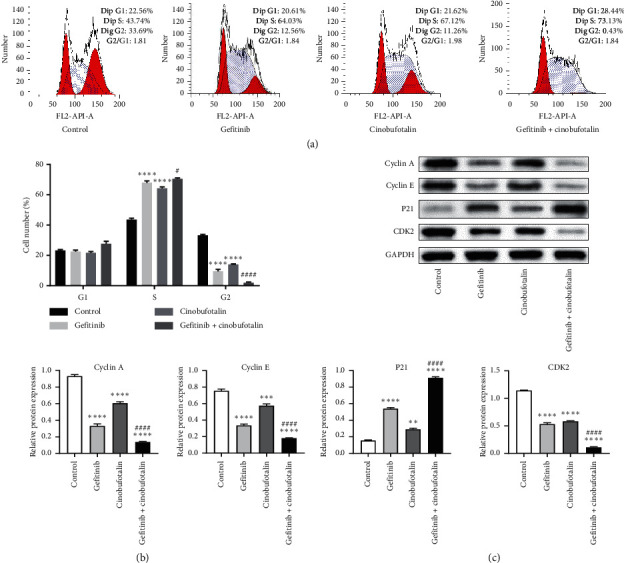
Combination of cinobufotalin and gefitinib inhibits A549 cell cycle in the S phase. (a) Flow cytometry data of cell cycle analysis. (b) Representative quantification of cell cycle analysis data indicating percentage of cells in given phases. (c) Western blot analysis of cell cycle control proteins in A549 cells in the presence of gefitinib, cinobufotalin, or cinobufotalin + gefitinib.  ^*∗∗*^*P* < 0.01,  ^*∗∗∗*^*P* < 0.001, and  ^*∗∗∗∗*^*P* < 0.0001 vs. the control group; ^#^*P* < 0.05 and ^####^*P* < 0.0001 vs. the gefitinib group.

Next, we performed cell cycle assay on A549 cells and found an increase of cell accumulation in the S phase, but a reduction in the G2 phase after treatment with gefitinib and cinobufotalin either alone or in combination (Figures 3(a) and 3(b)). Besides, the cell number in the S phase of the gefitinib + cinobufotalin group was higher than that of the gefitinib group (*P* < 0.05). As expected, the cell number in the G2 phase in gefitinib combined with the cinobufotalin group was significantly lower than that in gefitinib and cinobufotalin alone groups (*P* < 0.0001). Then, Western blot was performed to investigate the cell cycle-related proteins. The combined therapy of gefitinib and cinobufotalin significantly downregulated the expression of cyclin A, cyclin E, and CDK2 (*P* < 0.0001) (Figure 3(c)). On the contrary, P21 expression in the A549 cells was upregulated after treatment with cinobufotalin plus gefitinib (*P* < 0.0001).

### 4.4. Cinobufotalin in Combination with Gefitinib Promotes ROS Production

**Figure 4:**
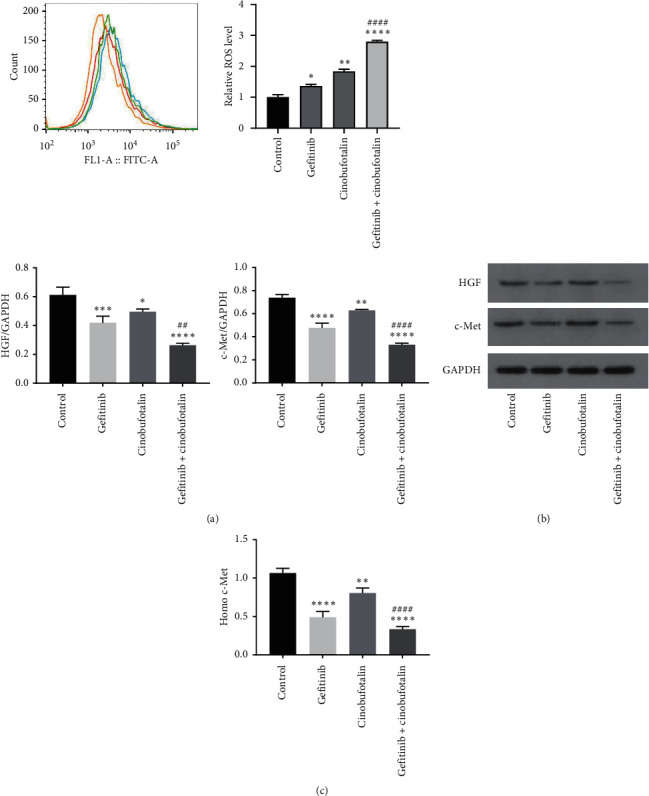
Combination of cinobufotalin and gefitinib induced ROS accumulation and suppressed the expression of HGF and c-Met. (a) ROS production was increased after combined treatment with cinobufotalin and gefitinib. (b) A549 cells were analyzed for HGF and c-Met protein expression by Western blot. (c) c-Met gene expression was determined by real-time quantitative PCR.  ^*∗*^*P* < 0.05,  ^*∗∗*^*P* < 0.01,  ^*∗∗∗*^*P* < 0.001, and  ^*∗∗∗∗*^*P* < 0.0001 vs. the control group; ^##^*P* < 0.01 and ^####^*P* < 0.0001 vs. the gefitinib group. The experiment was repeated three times.

To further confirm the involvement of ROS during cinobufotalin combined with gefitinib treatment, ROS generation was analyzed in A549 cells via DCFH-DA staining, followed by flow cytometry. As shown in Figure 4(a), compared with the control group, the ROS production in combination with the combined gefitinib group was obviously higher than that in cinobufotalin alone or gefitinib alone (*P* < 0.0001).

### 4.5. Cinobufotalin in Combination with Gefitinib Suppressed HGF and c-Met Expression

The underlying mechanism that resulted in the superior effect of gefitinib combined with cinobufotalin was evaluated. Treatment with cinobufotalin 0.5 mg/mL or gefitinib 40 *μ*mol/L alone or in combination (cinobufotalin + gefitinib group) significantly inhibited HGF/GAPDH and c-Met/GAPDH ratios to 0.4765 and 0.62857, respectively (Figure 4(b)). Compared with the gefitinib group, the protein levels of HGF and c-Met were further declined in the combination group (*P* < 0.01 and *P* < 0.0001, respectively). QRT-PCR analysis was performed to detect gene amplification of c-Met in A549 cells and found a significant reduction of c-Met gene amplification in the combination group (Figure 4(c)).

## 5. Discussion

The most notable finding of this study was that cinobufotalin combined with gefitinib enhanced A549 cell apoptosis and inhibited cell viability and cell cycle by downregulating HGF and c-Met protein expression. This finding fills the gap in the impact of combination of cinobufotalin and gefitinib on lung cancer. Targeted therapy has been a study focus in the field of cancer in the past decade. In the molecular biology research of NSCLC, EGFR mutation is a molecular target of general concern in medical research, and EGFR-TKIs such as gefitinib and erlotinib have been widely applied in clinical practice [13–15]. Although gefitinib has a certain beneficial function on human malignancy, it can also result in greater toxicity by killing normal cells. Moreover, the median time for many patients to develop resistance to gefitinib is about 10 months [16]. Therefore, it is of great urgency to find out a novel strategy to delay or overcome the acquired resistance to gefitinib.

Cinobufotalin is an animal-derived drug for the treatment of human malignancies. Qian et al. have implied that cinobufotalin regulates the expression of apoptosis-related protein Mcl-1 and invasion-related proteins (E-cadherin, MMP9, and Snail) by inhibiting c-Met expression in gallbladder cancer [17]. In addition, cinobufotalin can suppress gastric cancer cell proliferation and promotes cell apoptosis [12]. Although several studies have suggested that cinobufotalin has the potential to be a novel anticancer agent, little is known about the role of cinobufotalin combined with gefitinib in the treatment of lung cancer. In the current study, A549 cells were treated with cinobufotalin alone and found that cinobufotalin dramatically impeded the cell viability in a dose- and time-dependent manner. Several studies have confirmed prominent therapeutic efficacy of cinobufotalin combined with chemotherapy for NSCLC, which is more effective than chemotherapy alone [18]. Consistently, A549 cell viability in the combination group was significantly lower than that of the gefitinib group, indicating that cinobufotalin played a certain synergistic effect in combination with gefitinib.

The results of annexin-V/PI staining and flow cytometry revealed that the apoptosis of A549 cells in the gefitinib + combination group was significantly higher than that in the single treatment group, which was further confirmed by TUNEL assay. Activated proapoptotic proteins such as Bax result in the release of the apoptogenic cytochrome-c from the mitochondrial membrane, causing caspase cascade activation [19]. In this study, Bax and caspase-3 expression was elevated in A549 cells treated with gefitinib plus cinobufotalin, while the expression of antiapoptotic protein Bcl-2 was downregulated. Notably, compared with the normal group, the cell cycle of the gefitinib group and the cinobufotalin group was blocked in the S phase, and the blocking effect of the combined group was significantly enhanced, thus delaying cell mitosis. Besides, we also noticed downregulated expression of cyclin A, cyclin E, and CDK2 and upregulated expression of cyclin-dependent kinase inhibitor P21 in the gefitinib + cinobufotalin group. A recent report described that cinobufotalin induced a ROS-dependent autophagy cell death in lymphoma cells [19]. We speculated that ROS might be involved in the mechanism of gefitinib combined with cinobufotalin to lung cancer cell apoptosis. To verify the idea, DCFH-DA staining was performed to evaluate the ROS generation, and results showed that the induction of cell apoptosis by the combined therapy was linked to the generation of ROS. These data demonstrated that cinobufotalin potentiated the sensitivity of A549 cells to anticancer drug gefitinib, thereby promoting cell apoptosis.

In recent years, studies have found that some patients develop primary resistance to gefitinib or acquired resistance to gefitinib within 8–10 months, affecting progression-free and overall survival [20, 21]. A study conducted by Lu et al. pointed out that gefitinib resistance might be related with the activation of downstream EGFR signaling pathways such as MAPK/c-Fos and AKT/Bcl-2; besides, c-Met overexpression could also greatly limit the EGFR-targeted therapy [22]. The c-Met protooncogene can be amplified by bypassing the inhibited EGFR-phosphorylated kinase pathway through the ERBB3-PI3K-Akt and MAPK-ERk1/2T pathways. The amplified c-Met avoids killing EGFR-TKIs and promotes the proliferation of cancer cells by promoting downstream signal transduction through bypass activation and ultimately leads to drug resistance of patients to EGFR-TKIs [23]. Additionally, lung cancer cells can secrete a variety of cytokines to promote the continuous secretion of HGF by peripheral fibroblasts, thus forming a positive feedback loop, leading to the infinite growth of cancer cells [24]. Cancer cells with high c-Met expression are more sensitive and invasive to HGF. c-Met amplification is independent of the EGFR pathway, and 10% of EGFR-TKIS resistance is completely caused by c-Met gene amplification [25]. Our results showed that the combination of gefitinib and cinobufotalin remarkably downregulated the protein expression of c-Met and HGF, which further suggested that cinobufotalin could prevent the vicious cycle of the HGF/c-Met pathway to a certain extent.

In tumor cell lines with c-Met gene amplification, the growth of tumor cells depends on the c-Met gene signaling pathway. However, there is a certain relationship between the acquired resistance of EGFR-TKIS and c-MET gene amplification in about 20% of tumor cell lines without c-Met gene amplification [26]. In this study, the amplification of c-Met gene was noticeably impeded in gefitinib and cinobufotalin groups, which was further blocked by the combined treatment of gefitinib and cinobufotalin. Overall, these findings suggested that cinobufotalin not only elevated the sensitivity of lung cancer to gefitinib but also inhibited gene amplification of c-Met to a certain extent. The main limitation of this research is that the pathway mechanism of gefitinib combined with cinobufotalin against lung cancer remains unclear, and we will further investigate its potential downstream pathway mechanism in the future.

## 6. Conclusion

Cinobufotalin combined with gefitinib suppresses A549 cell viability and promotes cell apoptosis by downregulating HGF protein expression and blocking c-Met gene amplification, indicating that cinobufotalin may delay the occurrence of gefitinib resistance in lung cancer cells. In summary, this study provides a novel idea for the future study of the treatment of gefitinib-resistant lung cancer with cinobufotalin.

## Figures and Tables

**Table 1:** Primer sequences.

Primer	Primer sequence (5′⟶3′)	Length (bp)
Homo GAPDH	Forward: 5 “- TCAAGAAGGTGGTGAAGCAGG -3”	115
	Reverse: 5 “- TCAAAGGTGGAGGAGTGGGT -3”	

Homo c-Met	Forward: 5 “- TTAGTCATCCCAATGTCCTC -3”	240
	Reverse: 5 “- CATCCAGCATACAGTTTCTT -3”	

## Data Availability

The data used and/or analyzed during the current study are available from the corresponding author upon request.
